# Cultural Influences in Women-Friendly Labor-Saving Hand Tool Designs: The Milk Churner Case

**DOI:** 10.1177/0018720815623146

**Published:** 2016-01-13

**Authors:** William S. Kisaalita, Abia Katimbo, Edison J. Sempiira, Dana J. Mugisa

**Affiliations:** University of Georgia, Athens; Smallholder Fortunes, Kampala, Uganda

**Keywords:** sustainability, low-resource settings, human factors, smallholder farmers, Uganda

## Abstract

**Objective:**

The aim of this study was to highlight the importance of culture in sustainable, labor-saving solutions design for women in low-resource settings.

**Background:**

One of the reasons behind the gender asset gap among Sub-Saharan African women is the higher labor burden these women face, making it difficult for them to produce for the home and markets. Hand tools are the simplest form and therefore the best first step to address this problem. But designing women-friendly (sustainable) hand tools calls for better understanding of the low-resource settings where these women reside.

**Method:**

A milk churner was redesigned using a human-centered (participatory) approach with groups of women from two dominant ethnolinguistic groups of Bantu and Nilotic of Uganda, and its usability was tested.

**Results:**

The churner reduced labor up to eightfold and has potential to expand the range of uses to include children and husbands due to its simplicity. Also, the churner significantly reduced undesirable health effects, like pain in knee joints. Based on the experience with the churner, a six-item “survival guide” is proposed to complement human-centered design guiding principles for facilitating the generation of solutions in low-resource settings.

**Conclusion:**

By paying great attention to culture in relation to human factors, a labor-reducing churner has been successfully introduced among Ugandan women. The ultimate goal is to make the churner available to female smallholder dairy-farming households throughout Sub-Saharan Africa.

**Applications:**

This study provides a survival guide for generating solutions to problems from low-resource settings.

## Introduction

The gender asset gap among Sub-Saharan African women is very well known (Quisumbing et al., 2014). One of the reasons behind this inequality is the higher labor burden these women face, in comparison to their male counterparts, to maintain their household. Labor-saving interventions have been proposed as a way toward enabling these women not only to produce for the home but for the market as well (Sims & Kienzle, 2006). By increasing incomes through increased productivity, these women are better prepared to adapt to climate change (higher incomes enable diversification to less vulnerable income-generating activities), and better well-being of their families is realized (Blümmel et al., 2015). Some form of mechanization is a natural approach to address this problem, and on the mechanization continuum, hand tools are the simplest form and therefore the best first step.

In our companion paper (Mugisa, Katimbo, Sempiira, & Kisaalita, in press), we observed that lack of hand tool fit is due not only to poor or absence of anthropometry knowledge but also to lack of fit within cultural practice. An excellent example of a hand-operated technology that failed in the marketplace due to lack of fit in cultural practices is the PlayPump (Borland, 2011). The PlayPump was designed to pump ground water to a storage tank, from which the water would be distributed to water-starved communities. The energy for pumping was to be supplied by children and/or women in the community while they “played” on the merry-go-round structure connected to the pump. One of the reasons for the failure of the solution, especially where children would be at school most of the day, was that it was not dignified for women to “play” on the merry-go-round structure (Borland, 2011).

Another technology victim of poor culture fit from western Africa was a rice thresher. In efforts to reduce the labor burden, the thresher was mounted on a bicycle-like structure for foot operation (Taiwo & Faborode, 2002). With the common female-appropriate attire in this region, the foot operation of the device exposed too much female thighs, inviting inappropriate attention as seen with women wearing miniskirts elsewhere in Sub-Saharan Africa (Vincent, 2008).

Why fail to come up with fitting solutions from the get-go? The answer to this question boils down to two overlapping responses: First, well-meaning designers or technology developers on many occasions come up with solutions first and then look for a problem, and second, even in cases when the problem inspires the design or technology solution, designers/developers may have a very poor knowledge of the context, especially cultural, in which the solution will operate (Kisaalita, 2015). This problem has been recognized not only in developing but also in developed countries. To minimize the hit-and-miss solutions, a human-centered design and/or systems thinking is being advocated. In a similar spirit, we have introduced our own “connect-the-dots” approach for low-resource settings (Kisaalita, 2015). As in our approach, other approaches (e.g., participatory design/research, the lead user approach, codesign, ethnography, contextual design, and empathetic design; Steen, 2012) are underpinned by four guiding principles: (a) involving users to better understand their practices, needs, and preferences; (b) organizing project iterations in conducting the research and generating and evaluating solutions; (c) searching for an appropriate allocation of function between people and technology; and (d) organizing multidisciplinary work (International Organization for Standardization, 1999).

When we consider designing for the underserved (e.g., female smallholder farmers) or low-resource settings, the literature from the aforementioned participatory or human-centered approaches is lacking in important nuances that are critical to generating solutions that are sustainable or become innovations. Here, we distinguish a solution from an innovation; a solution becomes an innovation when it is widely adopted, probably in multiple communities or regions, which is an indication of sustainability. We use the term *women-friendly* to express three solution attributes that are prerequisites to sustainability, that is, economic viability (for solutions like hand tools used in income-generation activities), ergonomic appropriateness, and cultural appropriateness. Hand tools’ designs for labor reduction or income increase, in low-resource settings, pass our “3p-Innovations” filter, comprising poverty alleviation, prosperity generation, and planet sustainability (no undesirable environmental impact). Solutions that require ergonomics input that pass the “filter” naturally link sustainability and ergonomics/human factors as described by Zink and Fischer (2013).

Creating a solution that will turn into an innovation involves making decisions. So what are the themes that heavily influence this decision making, in relation to the four human-centered design guiding principles presented earlier? Guiding Principles 1 (better understanding of practices), 2 (project iterations), and 4 (multidisciplinary design teams) do not present any unique challenges that are specific to underserved or low-resource settings. But Guiding Principle 3 (people and technology function) is where expansion into more specific nuances of the principle may be helpful to design teams working on sustainable solutions (innovations). Our solution foci are hand tools for female smallholder farmers of Sub-Saharan Africa to reduce labor and/or increase income. In this paper, we have used a hand-operated milk churner, which separates butter fat from fermented milk or cream for ghee making (Sempiira, Katimbo, Mugisa, & Kisaalita, 2015), to first highlight the importance of culture in sustainable labor-saving solutions designs for women in low-resource settings and, second, to extend human-centered or participatory design Guiding Principle 3 (people and technology function) with nuanced practical “survival guide” themes, based on our experience with design iterations of the churner. We use two ethnolinguistic groupings of Sub-Saharan Africans (Bantu and Nilotic) as a surrogate for distinct cultural settings and/or physical characteristics. However, we recognize up front that although this grouping may be an oversimplification in some situations, it is a reasonable first step. Ghee is a yellow, semisolid, grain-textured class of clarified butter that originated in India and is commonly used in South Asia and many parts of Africa.

## Materials and Methods

### Hand Tool and Methodology

**Figure 1.**
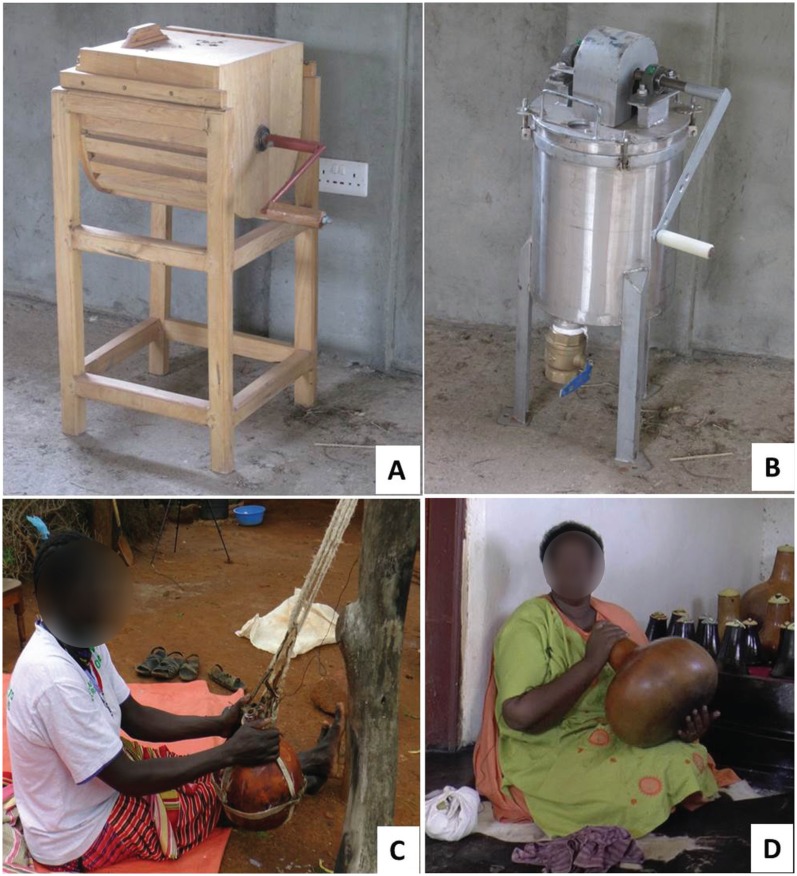
(A) First- and (B) second-generation churners in comparison to traditional churning styles in a gourd by (C) Nilotic (Jie tribe of northern Uganda) and (D) Bantu (Hima tribe of western Uganda).

As suggested in Guiding Principle 1, we initially studied the traditional ghee-making process, of which milk churning in a gourd (a dried fruit shell of the plant *Lagenera peucantha*) to separate butter fat (Figures 1C and 1D) is the most labor-intensive. The results of the traditional ghee-making process, in which churning was identified as an opportunity for intervention, are reported in a separate paper (Sempiira et al., 2015). The first-generation churner (Figure 1A) had been made earlier without the detailed study of the traditional ghee-making process and with little or no input from the women users (Muyanja, Kawongolo, & Kisaalita, 2009). Efforts to commercialize the churner failed. We speculate that this failure was due to the fact that it was not women-friendly. Reengineering the failed churner solution to make it women-friendly provided an opportunity to highlight the role of culture in women-friendly labor-saving hand tool design.

**Figure 2.**
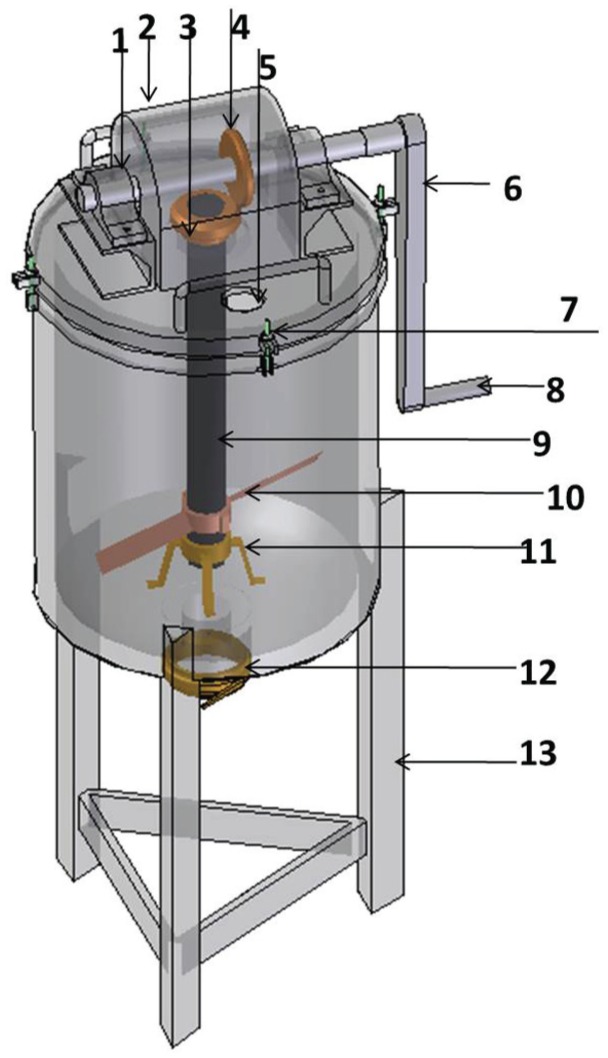
Isometric drawing of the churner (not to scale) showing all the components: (1) bearing bracket, (2) gear mechanism housing, (3) shaft bevel gear, (4) drive shaft bevel gear, (5) glass viewing window, (6) crank arm, (7) top plate fastener, (8) crank handle, (9) shaft, (10) baffles, (11) shaft bearing support and bush, (12) discharge (valve not shown), and (13) support.

Both the traditional ghee-making process study and the feedback from usability studies of the first-generation churner informed the design of the second-generation churner (Figures 1B and 2). The usability studies with the first-generation churner also enabled the development of a survey instrument (Supplementary Figure S1) with which to conduct second-generation churner usability studies, especially with respect to how women-friendly it was. With the first-generation churners, subjects who were identified as described later were introduced to the churner by training them on how to use it, leaving it with them for approximately 1 month, and coming back to monitor or listen to their suggestions (in focus group discussion) on how the product could be reengineered or improved to better serve them, in focus group–like discussions. The focus groups were restricted to only the participants who used the churner. The six questions used to drive the focus group conversation were as follows: (a) How many times did you use the churner? (b) What did you like most about the churner and why? (c) What did you not like about the churner and why? (d) In what ways should we change/reengineer the churner to best serve you? (e) Do you have any other suggestions/comments not covered in this conversation? (f) What questions do you have for us?

The second-generation churner was introduced to the subjects in a manner similar to the first-generation unit, with the exception that in this case, in addition to focus group discussions, a survey instrument was used. In all regions, the questionnaire was administered in the local language on a one-to-one basis, with the research assistant asking and recording the responses from the participant. This method was possible because the research assistants were fluent in the local languages. The exception was Kotido district; an interpreter was hired and was first familiarized with the context of the questions. The same interpreter was used throughout the study.

### Subjects

With the first-generation churner, female smallholder dairy farmers along the cattle corridor of Bantu and Nilotic ethnolinguistic groups were recruited for the study. The five locations included Ngoma village/town in Nakaseke district (Kabendera and Mukabashanbo churner employees), Nyamilinga and Kabuye villages in Kiboga district (Abesiga Mukama and Ikamiro groups), Kanyaryeru village in Kiruhura district (Kanyaryeru women group), and Kotido in Kotido district (Etiyata Kapei group). More details of these locations have been previously reported by Mugisa et al. (in press). Based on lessons learned from this first participatory engagement, a second-generation churner was designed (Figure 2). For usability, three of the aforementioned groups were dropped to accommodate three new groups. The rationale was to make sure the feedback we were getting from the old groups was not biased due to a sense of technology ownership established through the participatory nature of the approach used. We kept the most-well-organized groups of Etiyata Kapei (Kotido) and Abesiga Mukama (Kiboga). Three new groups were recruited from the same two ethnolinguistic groups. The three new groups included St. Anne women (Kotido location), Kiduduma women (Mubende location), and Abagambakimwe Development Association (Nakasongola location). A summary of the number of women participants in each group is presented in Table 1. Supplementary Figure S2 is included for a map showing the cattle corridor and the locations of all the groups that participated in usability studies of the second-generation churner.

**Table 1:** Summary of Participant Women Groups

Group Name and Location	First-Generation Churner Study Participants (Old)	Second-Generation Churner Study Participants (New)	Ethnicity
Kabendera and Mukabashambo employees (Ngoma town, Nakaseke district)	21	—	Bantu
Abesiga Mukama (Nyamiringa village, Kiboga district)	15	7	Bantu
Ikamiro (Kabuye village, Kiboga district)	12	—	Bantu
Kanyaryeru (Kanyaryeru village, Kiruhura district)	9	—	Bantu
Etiyata Kapei (Losilanga village, Kotido district)	32	7	Nilotic
Kiduduma (Kiduduma village, Mubende district)	—	0	Bantu
Abagambakimwe (Kafu village, Nakasongola district)	—	6	Bantu
St. Anne (Losilanga village, Kotido district)	—	14	Nilotic

*Note*. Data are number of women who participated in the focus group discussions. The labels *old* and *new* are used in reporting results to signify groups that participated in both first- and second-generation and only second-generation studies, respectively.

## Results and Discussion

### Lessons From First-Generation Design Usability and How They Informed the Second-Generation Design

The feedback/lessons from women users of the first-generation churner are shown in Table 2. We ignored ethnolinguistic classification of the participants to enable the design of a second-generation churner that will be “sensitive” to cultural differences. Every suggestion/comment was recorded. The results of the discussions of each of the points are presented, and in the comments section, thoughts and reflections solely from the design team (coauthors of this paper) are presented.

**Table 2:** Lessons Learned or Feedback From First-Generation Churner Usability Focus Groups (see Table 1) Toward Redesigning to Make Women-Friendly (Ergonomically and Culturally Appropriate)

Lesson/Feedback	Actions Discussed	Comments
1. Redesign crank/handle—crank is too long and handle diameter too small.	Conduct anthropometric study to size the crank/handle appropriately.	Is there significant difference in anthropometry measures between Nilotic and Bantu? Yes, different regions will require different sizes. No, one size will fit all.
2. Lower the churner height.	Conduct anthropometric study to determine the most suitable churner height. Be mindful of the differences in traditional churning posture between the two cultural groups.	Nilotic (Kotido location) prefer standing posture. Bantu (Mbarara, Ngoma, and Kiboga locations) prefer sitting posture.
3. Is it possible to make a driving mechanism that is low-speed input (low churning rpm), but high-speed output (high baffles shaft rpm)?	Consider mechanisms like bevel gear.	The bevel gear mechanism will work best, but what is the best gear ratio?
4. Can you make it foot operated as opposed to hand operated, like a sewing machine?	Hold on implementing this suggestion till the third-generation design, if still needed.	Implementing this suggestion will complicate the design from a cost and cultural viewpoints. The Bantu women wear clothes that cover them all the way to their ankles. A bicycle foot-operated mechanism might present a problem of need to change dressing, for example to pants, a “radical” change from traditional dress, etc.
5. Warping of the wood is causing poor sealing of the cover, subsequently causing spillage during churning.	What about using well- seasoned wood? What about making it out of metal or metal/wood combination?	Metal will make the device very expensive, but it all depends on the increase in income resulting from increased productivity.
6. Make it easier to completely remove the discharge by placing the drain at the bottom and on the crank side of the churner for ease of operation (operator working from the same position).	The discharge point can easily be placed at the bottom of the chamber.	No comment
7. The wooden stand should be replaced with a metallic one or the wood covered with metallic plates, or wood treated with anti-termite agent; in the present form, if left in one place for a while, termites will attack the wood.	Will be easily implemented with the second-generation design.	No comment
8. It seems to require more energy to churn in comparison to the traditional gourd.	The bevel gear mechanism mentioned in Item 3 will solve this problem.	A contribution to this high-energy requirement was partly due to poor bearings used in the first-generation design.
9. Conflicting feedback on churner capacity; some suggest increasing and others suggest reducing the capacity.	Maintain the current capacities of 20 and 10 liters. It will be convenient if the same design can accommodate both capacities, unlike in the current design whereby these are two different products.	The most common gourds handle 5 liters, fewer handle 10 liters, and those that can handle 15 liters are rare. Fifteen-liter gourds are mechanically weak and do not survive the traditional churning process long without breaking.
10. In the current configuration, it is difficult to remove the formed butter fat. Use gourd-like material to avoid butter sticking to the walls.	Increase the discharge diameter and flow the discharge through a sieve to catch small butter particles. Consider “butter-repelling” material coating on the wooden shaft and baffles.	The butter fat also seems to have an affinity with the wood material of the shaft and baffle and is hard to remove completely.
11. The shaft and baffles are difficult to assemble and disassemble.	Design the second-generation churner with assembling and disassembling of both the shaft and baffles in one operation.	This design will also make both the butter removal and cleaning easier.
12. Beautify the churner to match the gourd and/or *byanzi* (ebony black wood milk storage containers) outside look.	Implement the requested look.	No comment
13. What about making it two-hand operated?	With the implementation of a bevel drive mechanism mentioned in Item 3, it may not be necessary.	This feedback was probably a result of needing a higher level of energy as pointed out under Item 8.
14. Experiencing back pain after long churning hours	Conduct anthropometric study to design a better-“fitting” or “women-friendly” churner.	It is possible this is a result of change in churning posture, engaging parts of the body that have not been engaged hitherto.
15. Can you think of an automatic churning machine?	Yes, we can, but how will you operate it without electricity? The income from the ghee is too small to support use of a fossil fuel electricity generator. We will stick to hand-operated for now.	The target uses are women smallholder farmers who are predominantly rural with no access to grid electricity. Much-larger-capacity electrically powered churners exist for commercial dairies, a different clientele.

In response to Lessons 1, 2, and 14, we conducted an anthropometric study (28 variables, previously recommended for agricultural workers; Dewangan, Kumar, Suja, & Choudhury, 2005) of a total of 89 women participants in the first-generation churner usability study. No strength studies were undertaken; the design team reasoned that the strength need (Lessons 8 and 13) was adequately addressed by the introduction of a beveled gear drive mechanism with a gear ratio of 6 (Lesson 3). Second, the wood-warping problem (Lesson 5) in a hot and wet climate would not go away with well-seasoned wood; use of wood was abandoned and stainless steel was adopted. To minimize cost, the stand (Lesson 7) was made of mild steel and the cover/drive mechanism was made of mild steel with a thin stainless steel plate and shaft in contact with the milk. Also butter fat does not stick to stainless steel like it does to wood, which addressed Lesson 10. Third, the stainless steel shaft/mixing baffle, drive mechanism, and cover plate were integrated into a single piece that is assembled or disassembled with the milk container in a single operation (Lesson 11). Fourth, the circular nature of the milk container made it natural to add a wide-valve discharge at the bottom (Lesson 6). The foot-operated and conflicting churner capacity (Lessons 4 and 9) were not addressed, as discussed in Table 1. However, the second-generation design affords churning 10 to 20 liters in a single device. Also, notice in Figures 1C and 1D, the women churn while they are seated; we provided a stool of appropriate height to maintain the seated postures.

The design team added a glass window feature on the cover plate, which did not come up in participatory discussions, to be able to observe droplets of forming butter without having to take off the cover plate with the drive mechanism. In the traditional churning process in a gourd, the women users can tell that butter has formed by listening to the sound made within the gourd while churning. Butter formation is confirmed by looking into the gourd to see if droplets of butter are visible on the gourd wall close to the opening. We speculate that although the sound will not be the same, these operators will learn to identify that moment with the stainless steel vessel. By adding the glass window, the design offers opportunity to validate the meaning of the change in sound without having to open the churner. For beauty (Lesson 12), the stainless steel look gave the second-generation churner the “advanced technology” look, as pointed out by some women. For this reason, we decided not to paint the churner with a gourd/*byanzi* color.

### Second-Generation Churner Women-Friendliness Testing Results

We did not administer the questionnaire to one of the five groups (Bantu, Mubende location) because the group did not use the churner, leaving only four groups for statistical analysis. The reason provided for the low interest was that the group did not receive any assurance—which we could not give—that we would find the market for the increased ghee volume as a result of using the churner. Interestingly, throughout our fieldwork, we heard this request of help with marketing of ghee almost everywhere we went. Through our analysis (Katimbo, Sempiira, Mugisa, & Kisaalita, 2015), we have shown that the per capita consumption of ghee in Uganda is an order of magnitude lower than a recommended target value, which is still low in comparison to India, if used as the model. What is needed is establishment of a streamlined ghee value chain, from rural producers to urban and peri-urban consumers.

The question we wanted to address first was whether there was generally a difference between the old and new groups, to have more confidence that the old groups are not positively biased. Given that the responses to the survey question were not normally distributed, we used the Kruskal-Wallis test that is not constrained by distributional assumptions. Based on a *p* value of ≤.05, there were significant differences among the four groups with the questions shown in Table 3. By examining the means from the responses, we can conclude that the source of the difference was responses from the Nakasongola group. In almost all cases, Nakasongola responses are diametrically opposite to the other three groups. This finding is not surprising; Nakasongola were different from the other groups in that the group was mixed gender, with fewer men owning the operation and the women as employees hired to churn. On the first day of training, the men got excited about the technology because they could operate the churning themselves, either because of reduced labor/increased volume or because the churner represented a gender role–changing technology, getting around the cultural norm of churning in a gourd being reserved for females. The women privately expressed fear of losing their jobs and, for most of them, their livelihoods. Further evidence in support of this observation is presented in Table 4 for pain area as a result of churning. Nakasongola were the only group that scored the churner with *very severe problem*. For further analysis, we felt comfortable leaving the Nakasongola group out.

**Table 3:** Survey Questions Where Differences Among the Four Groups Were Found Based on *p* Values ≤.05

		Response Means			
Question	*p*	Kotido New (*n* = 14)	Kotido Old (*n* = 7)	Kiboga Old (*n* = 6)	Nakasongola New (*n* = 6)
1.3. Comfort with discharge removal mechanism^a^	.0375	1.28	1.14	1.14	3.00
1.5. Comfort with input effort^a^	.0311	2.797	1.43	1.861	2.33
1.6 Comfort with single-hand operation^a^	.0188	1.71	1.29	1.88	2.17
2.1M. pain level in/on hand^b^	.0011	1.79	1.00	1.43	3.50
2.2M. pain level in/on chest^b^	.0094	1.93	1.00	1.57	3.33
2.3M. pain level in/on back^b^	.0091	1.64	1.00	1.86	3.17
2.4M. pain level in/on palms^b^	.0006	1.64	1.00	1.57	4.00
2.4G. pain level in/on palms^b^	.0162	3.14	3.00	1.57	1.83
4. Enough machine capacity^a^	.0041	1.79	1.43	2.00	3.17
5. Machine replacing the gourd^a^	.0057	1.71	1.14	1.86	2.67
6. Using the machine instead of the gourd in the future^a^	.0007	1.43	1.14	1.71	2.67

*Note*. The labels *old* and *new* are used in reporting results to signify groups that participated in both first- and second-generation and only second-generation studies, respectively. M = machine (second-generation churner); G = gourd.

a Responses were 1 = *strongly agree*, 2 = *agree*, 3 = *neutral*, 4 = *disagree*, 5 = *strongly disagree*.

b Responses were 1 = *no problem*, 2 = *slight problem*, 3 = *moderate problem*, 4 = *severe problem*, 5 = *very severe problem*.

**Table 4:** Summary of Other Pains Identified and Their Levels: Questions 2.5M and 2.5G

Location and Group Type	M Pain Area (Pain Level)	G Pain Area (Pain Level)
Kotido old group		Waist (3)
Kotido new group		
Kiboga old group	Shoulder joint (4) and knee joint (1)	Waist (5) Knee joint (4)
Nakasongola new group	Shoulder joint (5) Waist (5)	Knee joint (4)

*Note*. M = machine (second-generation churner); G = gourd. The labels *old* and *new* are used in reporting results to signify groups that participated in both first- and second-generation and only second-generation studies, respectively. Each entry row represents mention by one participant, and the numbers in parentheses represent pain level as follows: 1 = *no problem*, 2 = *slight problem*, 3 = *moderate problem*, 4 = *severe problem*, 5 = *very severe problem*.

The other concern we had was whether the old groups were too optimistic in the opposite direction to Nakasongola. With the exception of scores of 1 in response to the machine pain question, the rest of the responses are in line with the other two groups. Even with pain questions, Kotido old-group scores are on the same side of the scale. For this reason, we felt comfortable with further analysis that included the Kotido old group.

We further compared the remaining three groups, two at a time, with the Wilcoxon Mann-Whitney test for better insights on the role of culture. As shown in Table 5, there were no major differences between the two cultures represented: Out of the 20 questions in the survey, only four (Questions 2.3M, 2.4M, 2.4G, and 4) turned out to be significantly different. The relative similarity between Kotido old and Kotido new groups was attributed to the positive bias in the old group, already pointed out. Therefore the two cultures (*Bantu–*Kiboga location and *Nilotic–*Kotido locations) either equally liked or equally disliked most aspects of the churner—this finding was contrary to our expectations.

**Table 5:** Survey Question Responses That Were Significantly Different in a Pairwise Comparison of the Three Groups With the Wilcoxon Mann-Whitney Test

Group Pair/Question	*p*	Response Means
Kiboga old group vs. Kotido old group		
2.3M. Pain level in/on back^a^	.0486	1.8571 vs. 1.0000
2.4M. Pain level in/on palms^a^	.0293	1.5714 vs. 1.0000
4. Enough machine capacity^b^	.0466	2.0000 vs. 1.4286
Kiboga old group vs. Kotido new group		
2.4G. Pain level in/on palms^a^	.0185	3.0000 vs. 3.1429
Kotido old group vs. Kotido new group		
1.5. Comfort with input effort^b^	.0242	1.4286 vs. 2.7857
2.1M. Pain level in/on hand^a^	.0436	1.0000 vs. 1.7857
2.2M. Pain level in/on chest^a^	.0284	1.0000 vs. 1.9286
2.3M. Pain level in/on back^a^	.0424	1.0000 vs. 1.6429
2.4M. Pain level in/on palms^a^	.0424	1.0000 vs. 1.6429
5. Machine replacing the gourd^b^	.0280	1.1429 vs. 1.7143

*Note*. M = machine (second-generation churner); G = gourd. The labels *old* and *new* are used in reporting results to signify groups that participated in both first- and second-generation and only second-generation studies, respectively.

a Responses were 1 = *no problem*, 2 = *slight problem*, 3 = *moderate problem*, 4 = *severe problem*, 5 = *very severe problem*.

b Responses were 1 = *strongly agree*, 2 = *agree*, 3 = *neutral*, 4 = *disagree*, 5 = *strongly disagree*.

In absence of culturally related differences in the foregoing analysis, we reasoned that it was possible the survey questions could not capture the cultural-difference construct. We turned to the focus group comments, presented in Table 6, together with response means for the three groups for all the questions in the survey with the exception of pain questions (Question 2). There are several observations from these comments. First, there were very few comments that were equally distributed between the two cultural groups (few Nilotic and Bantu together on one comment), lending support to the hypothesis that culture matters, but we were not able to measure the construct with our survey questions. However, this is an association and not causality. Second, the churner is a successful hand tool and will easily replace the gourd, as the response means and comments to Questions 5 and 6 suggest. Third, the response to efforts/energy was mixed. We think that there were women who were expending more energy than was needed, a problem we can adequately address with better training. Fourth, the churner reduces labor when fully used (20 liters, fourfold for a single churn in comparison to the 5 liters that are typically churned in the gourd). If 20 liters are churned twice a day, an eightfold reduction in labor or increase in productivity can be realized.

**Table 6:** Mean Scores and Associated Discussion Comments for Survey Questions 1 (Churner Physical Characteristics), 3 and 4 (Productivity), and 5 and 6 (Churner Preference Over the Gourd)

Question	Kotido New	Kotido Old	Kiboga Old	Comments From Focus Group–Like Discussions^a^
1.1. Height	1.93	2.14	1.86	Reduce cylinder height and make it round as in the gourd (N) Height is OK (B, B) When seated on the stool, the pain I get from sitting on the floor with the gourd disappears (B)
1.2. Crank/handle	1.79	2.29	1.71	Grip length is small if one wants to use both hands (B) Change the grip material to something soft (B)
1.3. Ghee/yogurt removal	1.29	1.14	1.14	Removal mechanism is fine (N)
1.4. Drive mechanism	2.29	1.14	1.86	Drive mechanism is soft reducing energy need (N)
1.5. Effort/energy input	2.79	1.43	1.86	Modify the machine to include a motor (N, N, N, N, B) You can churn slowly to conserve energy (N) Does not require so much energy; the whole process is like playing with a kid/baby (N, N, B) Effort required is too much; machine depletes energy from the body (B, B, B)
1.6. Single-hand operation	1.71	1.29	1.86	Can even alternate the stool to use left or right, whoever is stronger and can use both hands (N, B)
3. Reduction in churn time	1.98	1.57	1.43	Machine takes same time even if the milk quantity is small (N, N) With good-quality milk, churning time is the same as with the gourd (N)
4. Enough machine capacity	1.79	1.43	2.00	When you have a full 20 liters, you can do it once, unlike the gourd (B, B)
5. Machine replaces gourd	1.71	1.14	1.86	Yes, solves problems of flies, rats, dogs stealing the gourd when forgotten outside and even human thieves (N, N, N) The only suggestion is that you find us a ghee market to motivate us to start producing in large quantities (B) It is not delicate; it can easily be operated by children, unlike the gourd, which can be touched only by “mature” women (B, B, B) Ghee quality is similar to that from the gourd (B)
6. Will use machine in place of gourd	1.43	1.14	1.71	Yes, because gourd makes the yogurt (discharge) bitter (N, N, N) Will continue using the machine (B, B, B) Can work for many years without repair or replacement (B)

*Note*. The labels *old* and *new* are used in reporting results to signify groups that participated in both first- and second-generation and only second-generation studies, respectively.

a N (Nilotic) or B (Bantu) at the end of each comment indicates the cultural source of the comment; the repetition of letters in parentheses indicates the number of times the comment came up.

The self-reported pain levels between gourd and churner use, presented in Table 7, show the churner to significantly reduce churning-related pain or discomfort. However, it should be pointed out that although the means for the Kiboga old group are higher, suggesting less pain from churner use, the statistical comparison did not show a significant difference (all *p* values were higher than .05). This finding may be due to differences in postures between the Bantu (Kiboga) and Nilotic (Kotido). Further studies with surface electromyography recordings to confirm the self-reported discomforts are needed.

**Table 7:** Comparison of Group Mean Self-Reported Pain Levels for Churner (M) and Gourd (G) Operation Using the Signed Rank Test

Group/Pain Area	M Mean Score	G Mean Score	*p*
Kotido new group			
Hand	1.79	2.86	0.0625
Chest	1.93	3.14	0.0098
Back	1.64	3.13	0.0039
Palm	1.64	3.14	0.0034
Kotido old group			
Hand	1.00	2.43	0.0156
Chest	1.00	2.71	0.0313
Back	1.00	3.00	0.0156
Palm	1.00	3.00	0.0313
Kiboga old group			
Hand	1.43	1.71	0.7500
Chest	1.57	2.14	0.3750
Back	1.86	2.43	0.5625
Palm	1.57	1.57	1.0000

*Note*. The labels *old* and *new* are used in reporting results to signify groups that participated in both first- and second-generation and only second-generation studies, respectively. Responses were 1 = *no problem*, 2 = *slight problem*, 3 = *moderate problem*, 4 = *severe problem*, 5 = *very severe problem*.

### Sustainability

The relation between the human factors and ergonomics used in this project and sustainability can be thought of in three distinct ways. First, the use of the resulting tool reduces pain/undesirable effects compared with the traditional approach. Second, because of ease of use, it can be adopted by many users beyond its tipping point, taking it from a solution to an innovation. Third, with widespread use, the increase in income supports many livelihoods, contributing in significant ways to local economies. The question of which one of these three ways is the dominant link is not relevant, because as presented earlier, they are linked and equally needed for sustainability as defined by Zink and Fischer (2013). The realized incomes endow the users with increased capacity to deal with climate change in addition to realizing better household well-being.

The question to grapple with next was how to supplement human-centered guiding principles, based on the experience in this project to increase the chances of a design team generating a solution with higher likelihood of turning into an innovation among underserved populations. As already pointed out, the emphasis in our case is labor-reducing tools for Sub-Saharan African women in very-low-resource settings (e.g., rural). We present six items in response to the question. The reader can think of the six items as a practical survival guide. We have confidence in the six items because it has been over 6 months since our second-generation churner has been in the field, and we can report that it is still in active use. We are working on transitioning the project to a business, by producing and marketing a batch of 500 units with a few modifications. This plan puts the churner solution on a trajectory to an innovation. The six “action” items are presented in the following paragraphs.

#### Item 1: Start with the local cultural, civic, and/or spiritual leadership for “blessing” the design project

In the literature on human-centered or participatory research/design, leaders are typically presented as important stakeholders or sources of information and are recruited to be members of the team. For example, in a capacity-strengthening system design on Solomon Island, Redman-MacLaren et al. (2012) reported having two East Kwaito chiefs on the solution-seeking team. In another study on social influences of young people’s sexual health in Uganda, Bell and Aggleton (2013) reported including religious and local clan leaders as participants. In these two examples, the objective was mainly to obtain contextual information and not “blessing” of the project. However, by agreeing to be part of the team, the blessing of the project by leadership was automatically recognized and understood by the community. The blessing we emphasize here is for situations in which there is no contextual value in having the leader as part of the team. Therefore by *blessing* we mean an understanding of what the project is about and possibly explaining its potential benefits to the community by someone who is respected and not a stranger to the community. Also, being introduced to the community by leadership that should have vetted the designers/researchers endows the team with credibility.

We have a caution to share under this item. On two occasions on this project, the local leaders we started with for the blessing assumed characteristics of brokers; they wanted to choose the participants for us, they wanted the churner to be tested at their facility under their watchful eye, and so on. The participants they provided were from a different demographic than the one we thought was ideal for the project. Essentially, they were rewarding their political supporters and/or relatives by getting them involved in the project. Also, by assuming the role of a broker for the project, they were looking to be seen as progressive and to score political points among the people they led. In both cases, we abandoned the locations. Trying to bypass the leadership and go directly to the community can be disastrous. For example, in a previous project in low-resource settings, the first author of this paper was introducing biogas technology using cow dung as the substrate. The leadership proposed to be provided with free domestic biogas plants in exchange for support of the project. The proposal was rejected and the next thing that we heard from reluctant community members was that the food cooked with biogas from the type of plant we were introducing smelled of cow dung—which was totally untrue. The project was moved to other locations.

#### Item 2: Make sure you have the relevant anthropometry data

In the first-generation churner design, the crank arm length, as well as the churner height, had been sized using information from Western anthropometric databases, primarily generated with U.S. Army and Air Force personnel. As shown in Table 1, the women in our study found the dimensioning unsatisfactory, which led us to question the relevance of the Western databases’ information to Ugandan women. As mentioned before, we took the time and measured 89 women from the two ethnolinguistic groups. Not only did we find that there were significant differences between the Ugandan and American women, but there were also significant differences between the two Ugandan ethnolinguistic groups. For example, the 5th percentile grip diameter inside for American women is 3.8 cm, whereas the values for the Nilotic (Kotido women) and Bantu (the rest of the Ugandan women measured) were 4.2 cm and 2.1 cm, respectively (Mugisa et al., in press). It is well established that grip diameter affects the grip strength (Radwin & Haney, 1996). Because of the relevance of such differences in the design of hand tools, we are calling for extensive studies to include more ethnolinguistic groups, representative of Sub-Saharan African women in general, and to ascertain if within the same ethnolinguistic group there may be important variations.

#### Item 3: Make sure you have the relevant strength data

The narrative of “women-friendliness” in the context of hand tools includes concepts of economic viability and ergonomic and cultural appropriateness. For farmworkers, Sims and Kienzle (2006) have interpreted the ergonomic concept to include “physical work capacity, comparative work intensity, how hard people can work, measurement of work load, concept of fatigue, avoidance or reduction of fatigue and its effects” (p. xviii). Although we did not make any strength measurements in this study, the issue of requiring more energy input was raised in first-generation usability studies. Relevant limits, especially for hand tools for the target population, should be known. Our experience with anthropometric measurements suggests that relying on Western databases may be misleading. The literature on this subject contains several studies on Indian women (Vyavahare & Kallurkar, 2012) but hardly any on Sub-Saharan African women.

#### Item 4: Be mindful of the solution being consistent with prevailing gender roles

Ghee making, of which the churning of fermented milk or cream in a gourd is a central activity, is considered a female activity in the cattle corridor of Uganda. A churner design for male operation would definitely not be female-friendly. As pointed out by William and Julian, cited by Minde (2015), “Gender roles are a set of social and behavioral norms that are generally considered to be appropriate for either a man or a woman in a social or interpersonal relationship.” Changes in social norms, economic standing, and applicable technology can individually or in any combination shift gender roles. We observed this dynamic in the Nakasongola location, where the group was of mixed gender; the men were the business owners, and the women were employees fulfilling the female churning role. The men got excited about the higher-capacity churner that they perceived to be a gender churning role changer. Realizing the potential of losing their jobs, the women grew less interested in the churner, to protect their incomes. The construction or deconstruction of gender roles is therefore possible, making the roles less uniform, as observed by Minde. If the interest of the design team and the participants is to maintain the gender role, the tool design has to reflect this desire and vice versa. When the introduction of the tool is of less interest to a segment of the users, it is imperative to know so the value of the feedback from the threatened individuals can be weighed in decision making accordingly.

#### Item 5: Be mindful of the relationship between solution adoption and the prevailing household decision-making process

We observed limitation in movement placed on women by their husbands or male partners among the Bantu but not the Nilotic. For example, we could meet with the Abesiga Mukama group only on Sunday after church but not any other day. A similar observation has been made by Jeckoniah, Mdoe, and Nombo (2013) in a study to map gender roles in the onion value chain in northern Tanzania (Bantu). We quickly learned to have our engagement coincide with prearranged group meetings, like business meetings. We speculate that in such scenarios, the decision to adopt a solution will predominantly rest with the husband or male partner, suggesting that engaging the husband or male partner at the beginning of the project, more so in Bantu than in Nilotic cultures, will facilitate solution adoption down the road.

#### Item 6: If asset ownership is important to success of the solution, devise strategies to overcome lack thereof

The question of what to do about low asset ownership as a deterrent to increased investment by women in income-increasing activities is a grand challenge. Our focus on hand tools to reduce the labor burden or to increase incomes is a response to the challenge. Traditionally, in both the Nilotic and Bantu, the cows belong to the husbands or male partners. The women are provided a fraction of the milk to make ghee for household use. Kimaro et al. (2013) studied control over dairy farming resources in the Arumeru district of Tanzania (Bantu) and found women to contribute more labor than men. But women who were not in a group did not have full access and control over the dairy-farming enterprise. Women in groups also participated more in household decision making. This knowledge may be helpful to a design team interested in identifying women participants more primed to take the solution to an innovation. We are deliberately working with women in groups for this reason.

We float the idea of developing validated instruments to capture the state of women with respect to each of the aforementioned items. For example, asset ownership can be established on a scale of 0 to 10, and if the tool to be developed depends on the woman having a particular asset or not, in the long run, a point on the scale can be established to inform decisions in either direction. Alternatively, it may be possible to use a single measure that is a surrogate for a number of items. For example, it is possible for a woman who scores high on Items 4, 5, and 6 to also score high on the well-established General Well-Being Schedule (Fazio, 1977).

## Concluding Remarks

By paying great attention to culture in relation to human factors, a labor-reducing churner has been successfully introduced among Ugandan women. Although the survey instrument was not able to reveal how important culture was in the development of the churner, focus groups with the women users were supportive of a possible role for culture. Based on the experience of human-centered churner design, a six-item survival guide is provided. Success in placing the churner at the center of a ghee value chain will further validate the survival guide.

## Key Points

- A human-centered design approach was used to successfully deliver a women-friendly hand tool (churner) for ghee making in Uganda.
- Difference in culture was associated with important design decisions in generation of sustainable labor-saving hand tools with the churner as a model.
- The experience inspired a practical survival guide of six items that complement human-centered design principles in the context of low-resource settings.

## Supplementary Material

Supplementary material
