# Supplementary material for: Cultural Influences in Women-Friendly Labor-Saving Hand Tool Designs: The Milk Churner Case
Source: Hum Factors. 2016 Jan 13;58(1):27–42. doi: 10.1177/0018720815623146 (PMC4768704; doi:10.1177/0018720815623146)
Supplement: Supplementary material [file suppl-material.pdf]

QUESTIONAIRE

INVESTIGATING ERGONOMIC AND CULTURRAL APPROPRIATENESS OF THE BUTTER CHURNER

SECTION 0: RECORDER IDENTIFICATION

NAME OF RECORDER:…………………………………………………………………

DATE:……………………………………………………………………………………….

SECTION 1: LOCATION DATA

DISTRICT:………………………………………….   SUBCOUNTY:……………………………………

VILLAGE:……………………………………..........

SECTION 2: GENERAL INFORMATION:

NAME OF RESPONDANT:…………………………………………………AGE:……………

GROUP NAME:………………………………………………………………………………

TRIBE: (circle one)

i) Munyarwanda (Bantu)
ii) Munyankole (Bantu)
iii) Jie (Nilotic)
iv) Others (specify)……………………

SECTION 1: REACTIONS AFTER LONG TERM MACHINE USE

1. I am comfortable with the machine …..(circle the most correct response)

**1.1 Height:**<br>1. Strongly agree<br>2. Agree<br>3. Neutral<br>4. Disagree<br>5. Strongly disagree	**1.2 Handle:**<br>1. Strongly agree<br>2. Agree<br>3. Neutral<br>4. Disagree<br>5. Strongly disagree
**1.3 Removal mechanism**<br>1. Strongly agree<br>2. Agree<br>3. Neutral<br>4. Disagree<br>5. Strongly disagree	**1.4 Driving mechanism**<br>1. Strongly agree<br>2. Agree<br>3. Neutral<br>4. Disagree<br>5. Strongly disagree
**1.5 Effort/energy input**:<br>1. Strongly agree<br>2. Agree<br>3. Neutral<br>4. Disagree<br>5. Strongly disagree	**1.6 Single-hand operation**<br>1. Strongly agree<br>2. Agree<br>3. Neutral<br>4. Disagree<br>5. Strongly disagree

2. The pain level on/in (mark the appropriate circle):

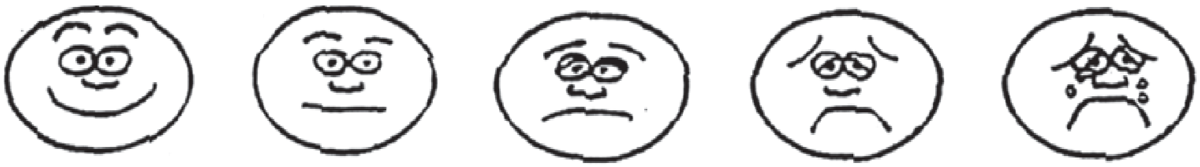

1 2 3 4 5

No problem | Slight problem | Moderate | Severe Problem | Very severe problem

Gourd	Machine
2.1G Hand 1 2 3 4 5	2.1M Hand 1 2 3 4 5
2.2G Chest 1 2 3 4 5	2.2M Chest 1 2 3 4 5
2.3G Back 1 2 3 4 5	2.3M Back 1 2 3 4 5
2.4G Palms 1 2 3 4 5	2.4M Palms 1 2 3 4 5
2.5G Others (specify)………………… ……. 1 2 3 4 5	2.5M Others (Specify)………………………… 1 2 3 4 5

3. There is a reduction in the churning time.
    1. Strongly agree
    2. Agree
    3. Neutral
    4. Disagree
    5. Strongly disagree

4. This machine capacity is enough.
    1. Strongly agree
    2. Agree
    3. Neutral
    4. Disagree
    5. Strongly disagree

5. This machine replaces the gourd.

    1. Strongly agree
    2. Agree
    3. Neutral
    4. Disagree
    5. Strongly disagree
6. In the future I will use this machine instead of a gourd.
    1. Strongly agree
    2. Agree
    3. Neutral
    4. Disagree
    5. Strongly disagree

SECTION 6:

Observation by researchers!

……
……
……
……
……
……
……
……
……
……
……
……
……
